# DNA methylation-mediated *FGFR1* silencing enhances *NF-κB* signaling: implications for asthma pathogenesis

**DOI:** 10.3389/fmolb.2024.1433557

**Published:** 2024-09-23

**Authors:** Minglu Meng, Yingjiao Ma, Jianguo Xu, Gao Chen, Roshan Kumar Mahato

**Affiliations:** ^1^ School of Public Health, Youjiang Medical University for Nationalities, Baise, China; ^2^ Faculty of Public Health, Khon Kaen University, Khon Kaen, Thailand; ^3^ Department of Respiratory Medicine, Affiliated Hospital of YouJiang Medical University for Nationalities, Baise, China; ^4^ Department of Laboratory Medicine, The People’s Hospital of Hechi, Hechi, China

**Keywords:** FGFR1, NF-ĸB, DNA methylation, asthma, 5-aza-CdR

## Abstract

**Background:**

Fibroblast growth factor receptor 1 (*FGFR1*) is known to play a crucial role in the pathogenesis of asthma, although the precise mechanism remains unclear. This study aims to investigate how DNA methylation-mediated silencing of *FGFR1* contributes to the enhancement of NF-κB signaling, thereby influencing the progression of asthma.

**Methods:**

RT-qPCR was utilized to assess *FGFR1* mRNA levels in the serum of asthma patients and BEAS-2B, HBEpiC, and PCS-301-011 cells. CCK8 assays were conducted to evaluate the impact of *FGFR1* overexpression on the proliferation of BEAS-2B, PCS-301-011, and HBEpiC cells. Dual-luciferase and DNA methylation inhibition assays were performed to elucidate the underlying mechanism of *FGFR1* gene in asthma. The MassARRAY technique was employed to measure the methylation levels of the *FGFR1* DNA.

**Results:**

Elevated *FGFR1* mRNA levels were observed in the serum of asthma patients compared to healthy controls. Overexpression of *FGFR1* in BEAS-2B cells significantly enhanced cell proliferation and stimulated NF-ĸB transcriptional activity in HERK-293T cells. Furthermore, treatment with 5-Aza-CdR, a DNA demethylating agent, markedly increased the expression of *FGFR1* mRNA in BEAS-2B, PCS-301-011, and HBEpiC cells. Luciferase activity analysis confirmed heightened NF-ĸB transcriptional activity in *FGFR1*-overexpressing BEAS-2B cells and BEAS-2B cells treated with 5-Aza-CdR. Additionally, a decrease in methylation levels in the *FGFR1* DNA promoter was detected in the serum of asthma patients using the MassARRAY technique.

**Conclusion:**

Our findings reveal a potential mechanism involving *FGFR1* in the progression of asthma. DNA methylation of *FGFR1* inactivates the NF-ĸB signaling pathway, suggesting a promising avenue for developing effective therapeutic strategies for asthma.

## 1 Introduction

Asthma is a heterogeneous and complex disease, characterized by diverse phenotypes and endotypes, each with distinct underlying mechanisms (Wenzel, 2012). These phenotypes and endotypes are influenced by a combination of genetic, environmental, and immunological factors (Lehto et al., 2019; BAJCOiP, 2019). Understanding the molecular basis of these variations is crucial for developing targeted therapeutic strategies.

Fibroblast growth factor receptor 1 (*FGFR1*) is a cytoplasmic protein-tyrosine kinase that belongs to the FGFR gene family (*FGFR1*–4). *FGFR1* is activated by binding with the essential fibroblast growth factor (bFGF) and is crucial for various cellular processes, including mitosis and differentiation. Recent research has highlighted the role of *FGFR1* signaling as a pivotal regulator of inflammation, implicating its involvement in numerous chronic inflammation-related disorders, such as cancer (Chen et al., 2020; Wang et al., 2020). Studies on lung cancer models have demonstrated that *FGFR1* promotes cancer progression and is linked to risk factors for respiratory conditions like bronchiolitis and chronic inflammation. Inhibiting *FGFR1* has been shown to downregulate the expression of genes associated with asthma pathogenesis, including SOX2, a marker of epithelial-mesenchymal transition (EMT), as well as the mesenchymal markers N-cadherin and Vimentin (Wang K. et al., 2018). Elevated levels of *FGFR1* expression in airway epithelial cells suggest its involvement in airway remodeling, a significant characteristic of asthma (Balasooriya et al., 2016; Tsai et al., 2018; Yuan et al., 2020). (Loffredo et al., 2017). Despite these findings, the precise role of *FGFR1* in bronchial epithelial cells and its mechanisms contributing to asthma pathogenesis remain inadequately elucidated.

The NF-κB signaling pathway regulates various multicellular developmental processes, including cell cycle progression, cell adhesion, inflammation, and angiogenesis (TJERCIWoS, 2009; El-Nagar and Elsisi, 2023; Chusongdam et al., 2024). Activation of the canonical pathway occurs through Toll-like receptors (TLRs) and proinflammatory cytokines, such as TNF-alpha and IL-1, leading to the activation of the IKKalpha-IKKbeta-NEMO kinase complex via TRAF complexes. This complex then phosphorylates and ubiquitinates IkB, releasing NF-kB dimers (such as p50-p65) to translocate into the nucleus and initiate the transcription of target genes. Negative feedback regulation is achieved through synthesizing IkB proteins and binding TNF receptor-associated factors (TRAFs) to inhibit NF-kB activity, thereby establishing a regulatory loop. In contrast, specific receptors and ligands, such as CD40L and BAFF, activate the non-canonical pathway, resulting in the phosphorylation of p100 by inhibitory IKKalpha, leading to its partial proteasomal degradation into p52. The RelB-bound p52 then translocates to the nucleus to modulate gene transcription (Pisani et al., 2022; Sun, 2017). Dysregulation of the NF-κB system has been implicated in various human diseases, notably inflammatory and fibrotic disorders. Extensive research has underscored the pivotal role of the NF-κB system in inflammation and immunity (Zhang et al., 2021). Lin et al. proposed a link between the NF-κB system and mucus hypersecretion, a hallmark feature of asthma (Lin et al., 2015). Moreover, studies have shown that activation of the NF-κB system contributes to airway remodeling and exacerbates neuroinflammation in a murine model of asthma (Kim et al., 2018). Our findings contribute to the understanding of asthma’s molecular complexity by suggesting that FGFR1 hypomethylation and the subsequent activation of NF-κB signaling may be associated with specific asthma endotypes, particularly those linked to airway remodeling and chronic inflammation.

Gene-environment interactions are pivotal in the development and progression of complex diseases, including asthma. Epigenetic mechanisms, such as DNA methylation, serve as critical mediators in these interactions by regulating gene expression in response to environmental stimuli. This dynamic interplay between genetic predisposition and environmental factors underscores the importance of epigenetics in understanding asthma pathogenesis (Potaczek et al., 2017). Given that epigenetic regulation, particularly DNA methylation, plays a significant role in asthma by modulating gene expression (Yang, 2019), we aimed to investigate the interaction between *FGFR1* and NF-κB signaling in bronchial epithelial cells. Specifically, we examined the effect of 5-Aza-CdR, a DNA methylation inhibitor, on this regulatory axis in BEAS-2B cells, following evidence of elevated *FGFR1* expression in asthma patients. Additionally, we assessed the clinical relevance of DNA methylation in regulating *FGFR1* expression in the serum of asthma patients.

## 2 Methods

### 2.1 Subjects and blood samples

Between January and April 2020, 43 asthma patients from the Affiliated Hospital of Youjiang Medical University for Nationalities were included in our study. A physician diagnosed these patients according to the criteria outlined in the Chinese guidelines for the prevention and treatment of bronchial asthma (2016 Edition). Serum samples (500 µL) were obtained from these patients prior to treatment. Additionally, 47 healthy donors matched in age, sex, and BMI were included as control subjects.

### 2.2 Cells and plasmids

Human normal lung epithelial cells (BEAS-2B cells), normal human bronchial epithelial cells (HBEpiC), and Primary Small Airway Epithelial Cells (PCS-301-011) were procured from the China Center for Type Culture Collection (Wuhan, China). The cells were cultured in Dulbecco’s modified Eagle’s medium/F12 (DMEM/F12) at 37°C in a 5% CO2 atmosphere. PCS-301-011 cells were maintained following the instructions in the Bronchial Epithelial Growth Kit (ATCC, USA). NF-κB luciferase reporter plasmids (NF-κB Luc) and pCMV-N-Flag plasmids were purchased from Yeasen Co., Ltd., Shanghai, China.

### 2.3 Cell transfection and overexpression of *FGFR1*

To upregulate *FGFR1* expression, reverse transcription-PCR (RT-PCR) was performed to amplify its cDNA sequences in BEAS-2B cells using specific primers: forward primer 5′TGGCACCCGAGGCATTATTT3′ and reverse primer 5′GTACAAGAAAGTTGGGCAGCG3'. The PCR-amplified cDNA was gel-purified and extracted using a QIA quick gel extraction kit. The *FGFR1* gene was cloned into pCMV-N-Flag vectors (Biovector Co., LTD, China). The resulting pCMV-N-Flag-*FGFR1* plasmid and empty vectors were used to transfect 5 × 105 sub-confluent HEK293T cells and virus skeleton plasmids. The HEK293T medium was centrifuged and sterile-filtered before using the viral supernatant to transduce BEAS-2B, HBEpiC, and PCS-301-011 cells for 48 h. Transformed colonies were selected based on ampicillin resistance on plates containing 100 μg/mL of ampicillin for 8 days. Surviving clones were further validated through Western blotting and Sanger sequencing.

### 2.4 RT-qPCR

Total RNA (100 ng) was extracted from serum samples, BEAS-2B cells, HBEpiC, and PCS-301-011 using the Plasma/Serum Circulating and Exosomal RNA Purification Kit (Amyjet Scientific Inc., China). Subsequently, 1 μg of total RNA was reverse-transcribed into cDNA with the ReverTra Ace qPCR RT kit (Toyobo, Japan) to detect *FGFR1* mRNA expression via a quantitative polymerase chain reaction. The relative expression of *FGFR1* mRNA compared to GAPDH was quantified using the comparative (2-ΔΔ) Ct method.

### 2.5 Western blot analysis

Total protein extracts from BEAS-2B, HBEpiC, and PCS-301-011 cells were obtained using RIPA lysis buffer and quantified with a BCA kit (Beyotime, China). Subsequently, 10 µg of protein samples were separated on a 10% SDS-PAGE gel and transferred onto a nitrocellulose membrane. Following blocking with 5% non-fat skim milk, the membranes were probed with primary antibodies: anti-flag (Cat#FNab03154, Fine Test, Wuhan, China, 1:1,000), anti-Ikba (Cat#FNab04198, Fine Test, Wuhan, China, 1:1,000), and anti-GAPDH (Cat#FNab03343, Fine Test, Wuhan, China, 1:1,000). Detection of signals was achieved using a western chemiluminescent HRP substrate (Cat#FNSA-0162/FNSA-0160, Fine Test, Wuhan, China, 1:1,000) and an enhanced chemiluminescence system.

### 2.6 Luciferase reporter assays

The NF-κB luciferase reporter plasmid was procured from QCbio Science and Technologies Co., Ltd., China and subsequently transfected into BEAS-2B, HBEpiC, and PCS-301-011 cells along with Pcmv-n-Flag-*FGFR1* plasmid or control vectors using Lipofectamine 3,000 Transfection Reagent (Invitrogen, L3000). Luciferase activity was measured 48 h post-transfection using the Promega Luciferase Assay System (Promega, United States).

### 2.7 DNA extraction and methylation assay

DNA was extracted from extracellular DNA in peripheral blood samples using a cfDNA Library Kit from Beijing Baiaolaibo Technology Co., Ltd., China. Quantification of DNA was performed using a NANODROP 1000. Methylation analysis of *FGFR1* at a concentration of 300 ng/μL was carried out by BioMiao Biological Technology (Beijing) Co., Ltd. The isolated DNA underwent sodium bisulfite treatment and PCR amplification using the PyroMark PCR Kit to enrich *FGFR1* sequences. The extracted extracellular DNA was then subjected to methylation. After SAP treatment (2 µg DNA), the samples (500 ng) were analyzed for targeted CpG island methylation using the MALDI-TOF MassARRAY system on a SpectroCHIP. The specific primers used were: 5′AGGAAGAGAGTTATTGTAGGTTGGAGATTTTTGGA3'; 3′CAGTAATACGACTCACTATAGGGAGAAGGCTACCCTTCTCTTCCTACAACCTAATC5'.

### 2.8 Statistical analysis

Prism 9.0 software facilitated data analysis. For data with normal distributions, parametric tests were conducted. Specifically, an unpaired *t*-test was used for two-group comparisons, while one-way ANOVA followed by Dunnett’s *post hoc* test was applied for multiple group comparisons. For non-parametric distributions, the Mann-Whitney U test was employed. For categorical or qualitative parameters, such as sex, comparisons were performed using the chi-square test. Statistical significance was designated at *p* < 0.05, with each experiment conducted at least three times in triplicate.

### 2.9 Ethical clearance

The Medical Ethics Committee of Youjiang Medical University for Nationalities obtained the ethical approval. (No.2017030501).

## 3 Results

### 3.1 Elevated expression of serum *FGFR1* in asthma patients

Our study observed no significant variances in sex, age, and BMI between the case and control groups. Nevertheless, upon adjusting for these factors, individuals with asthma exhibited 2.52 times higher levels of *FGFR1* mRNA compared to the control group, as indicated in Table 1.

**TABLE 1:** Baseline characteristics of the patient: Cases (n = 47) and control (n = 43).

Characteristics	Control n (%)	Case (%)	Crude OR	Adjusted OR	95% CI	*p*-value
Sex						0.413
Female	21(53.85)	18(46.15)	1	N/A	N/A	
Male	22(43.14)	29(56.86)	1.54	1.43	0.61 to 3.35	
Age						
Mean ± SD	49.79 ± 2.05	47.62 ± 2.54	−2.17	0.99	0.97 to 1.02	0.544
MBI						
Mean ± SD	22.49 ± 0.52	21.77 ± 0.39	−0.72	0.93	0.81 to 1.07	0.335
*FGFR1* mRNA levels						
Mean ± SD	0.98 ± 0.03	3.50 ± 0.09	2.52	2.52	2.32 to 2.73	<0.001

BMI: body mass index.

### 3.2 *FGFR1* promotes cell proliferation and NF-κB activity *in vitro*

**FIGURE 1:**
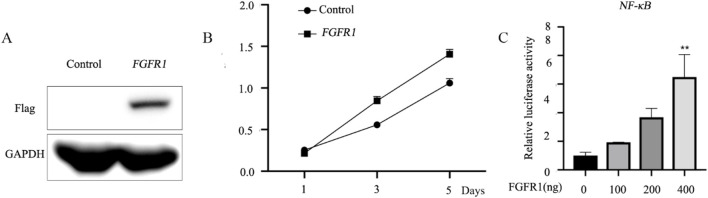
*FGFR1* promotes the cell proliferation and NF-κB activity *in vitro*. **(A)** Western blot analysis of Flag-*FGFR1* protein expression in BEAS-2B cells transfected with *FGFR1*-overexpressing plasmids and the corresponding empty control. **(B)** Highly expressed *FGFR1* lowered the cell viability of BEAS-2B cells. **(C)** Luciferase reporter assays were used to examine the NF-κB -mediated luciferase activity in BEAS-2B cells after incubated with (0, 100, 200, 400 ng) *FGFR1*-overexpressing plasmid and NF-κB reporter plasmids. ^**^represents as *p* < 0.05, *p* < 0.001 and *p* < 0.00001, respectively.

The recent study indicates that upregulated *FGFR1* expression in H1581 and DMS114 cells induces epithelial-mesenchymal transition (EMT), potentially contributing to asthma pathology. Our data showed that *FGFR1*-overexpression vectors were constructed and transfected into BEAS-2B cells, with overexpression confirmed via Western blotting (Figure 1A). Cell Counting Kit-8 (CCK8) assays were conducted to assess BEAS-2B cell proliferation, revealing that increased *FGFR1* expression enhanced cell proliferative capacity *in vitro* (Figure 1B). Furthermore, demethylation of *FGFR1* was linked to heightened NF-κB activity, as demonstrated by dual-luciferase assays (Figure 1C).

### 3.3 5-Aza-CdR increases *FGFR1* mRNA expression and activates the NF-ĸB signaling pathway

**FIGURE 2:**
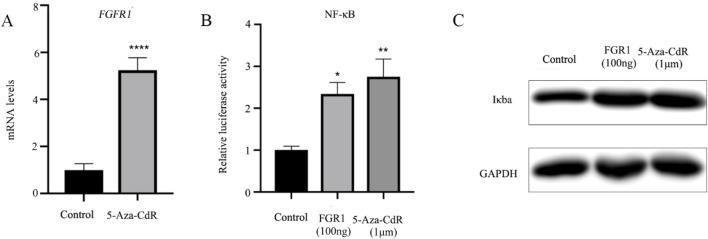
5-Aza-CdR increases the *FGFR1* mRNA expression and activates the NF-κB signaling pathway. **(A)** RT-qPCR examined *FGFR1* mRNA levels in BEAS-2B cells incubated with 1 um 5-Aza-CdR for 48 h or not. **(B)** Luciferase-based NF-Kb was determined in BEAS-2B cells incubated with 1 um 5-Aza-CdR for 48 h or transfected with *FGFR1*-overexpression plasmids. **(C)** Western blot detection of Iκba protein expression in BEAS-2B cells incubated with 1 um 5-Aza-CdR for 48 h or transfected with *FGFR1*-overexpressing plasmids. ^*^, ^**^, ^***^ represents as *p* < 0.05, *p* < 0.001 and *p* < 0.00001, respectively.

Numerous studies have confirmed the involvement of DNA methylation in the pathogenesis of asthma, specifically through gene silencing via CpG island methylation in the gene promoter (Hudon Thibeault and Laprise, 2019; Reese et al., 2019; Xu et al., 2018). The methyltransferase inhibitor 5-Aza-CdR has been shown to induce DNA hypomethylation, leading to increased gene transcription. This study, BEAS-2B cells were treated with 1 µm 5-Aza-CdR for 24 h, followed by RT-qPCR analysis of *FGFR1* mRNA levels. The results, depicted in Figure 2A, revealed an approximately five-fold elevation in *FGFR1* expression in BEAS-2B cells post 5-Aza-CdR treatment. Subsequently, the relative luciferase activity of NF-ĸB in BEAS-2B cells was assessed after treatment with 5-Aza-CdR. Overexpression of *FGFR1* in BEAS-2B cells resulted in a notable increase in NF-ĸB transcriptional activity, with a more pronounced effect observed in cells treated with 5-Aza-CdR (Figure 2B). Western blot analysis was then conducted to assess IκBα protein expression, showing that both elevated *FGFR1* expression and 5-Aza-CdR treatment led to increased IκBα expression levels (Figure 2C).

### 3.4 *FGFR1* promotes the growth of HBEpiC and PCS-301-011 cells

**FIGURE 3:**
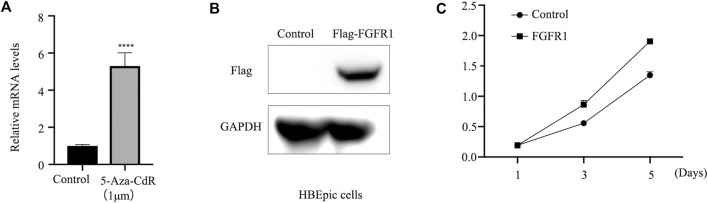
*FGFR1* promotes the growth of HBEpiC cells. **(A)** RT-qPCR examined *FGFR1* mRNA levels in HBEpiC cells incubated with 1 um 5-Aza-CdR for 48 h or not. **(B)** western blots examined *FGFR1* protein levels in HBEpiC cells transfected with *FGFR1*-overexpressing plasmids. **(C)** CCK8 assays demonstrating the cell proliferation, ^****^ represents *p* < 0.001.

The expression of *FGFR1* was assessed in HBEpiC and PCS-301-011 cells following treatment with 5-Aza-CdR. RT-qPCR analysis revealed increased *FGFR1* levels in HBEpiC cells post 5-Aza-CdR treatment (Figure 3A). Overexpression of *FGFR1* (Figure 3B) was associated with enhanced cell viability (Figure 3C).

**FIGURE 4:**
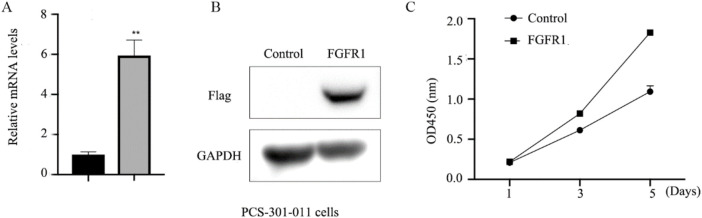
*FGFR1* promotes the growth of PCS-301-011 cells. **(A)** RT-qPCR examined *FGFR1* mRNA levels in PCS-301-011 cells incubated with 1 um 5-Aza-CdR or not for 48 h. **(B)** western blots examined *FGFR1* protein levels in PCS-301-011 cells transfected with *FGFR1*-overexpressing plasmids. **(C)** CCK8 assays demonstrating the cell proliferation, ^**^ represents *p* < 0.001.

To further elucidate the role of *FGFR1* in asthma, the impact of 5-Aza-CdR on *FGFR1* expression in PCS-301-011 cells was investigated. As depicted in Figure 4A, treatment with 5-Aza-CdR led to upregulation of *FGFR1* expression. Subsequently, *FGFR1* was overexpressed in PCS-301-011 cells (Figure 4B). CCK8 assays demonstrated that *FGFR1* overexpression promoted the proliferation of PCS-301-011 cells (Figure 4C).

### 3.5 *FGFR1* DNA methylation in the serum of asthma patients

**FIGURE 5:**
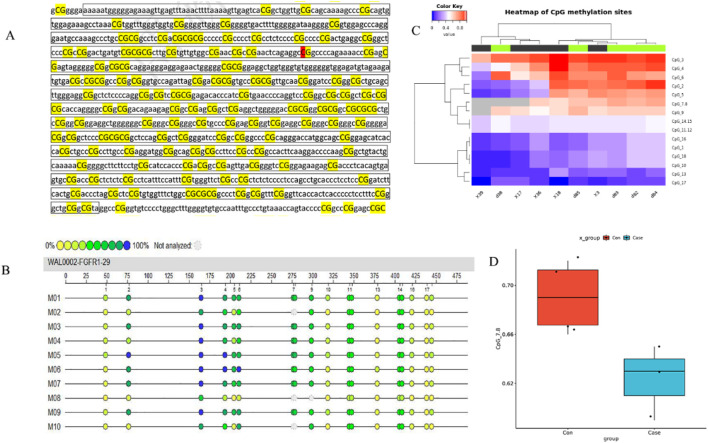
Declined methylation of *FGFR1* promoter CpG island in asthma patients.^**^ **(A)** The CpG island regions are contained in the *FGFR1* promoter. **(B)** Heatmap of BRCA methylation differences in ten independent samples (5 cases and five age-, sex-, race-, and BMI-matched healthy controls). **(C)** Differentially methylated sites in the *FGFR1* promoter. **(D)** Compression of *FGFR1* methylation in cases (n = 43) and healthy controls (n = 43).

The impact of 5-Aza-CdR on *FGFR1* expression was assessed by investigating *FGFR1* DNA methylation in five asthma cases and five healthy controls. Only one CpG island was initially anticipated in the *FGFR1* DNA promoter using the CpG Island Searcher tool (accessible at http://www.cpgislands.com) (Figures 5A,B). Subsequently, genomic DNA from *FGFR1* was isolated from serum samples and analyzed using MassARRAY spectrometry to quantify *FGFR1* DNA methylation, revealing twelve CpG sites (Figure 5C). Upon examination, a reduction in methylation levels was observed in asthma patients compared to healthy controls (Figure 5D).

## 4 Discussion

Higher levels of *FGFR1* mRNA were observed in the serum of asthma patients compared to healthy controls in our study. Overexpression of *FGFR1* notably enhanced the proliferative capacity of BEAS-2B cells and triggered NF-ĸB transcriptional activity. Our investigation into the DNA methylation mechanism of *FGFR1* expression revealed that treating BEAS-2B cells with 5-Aza-CdR led to increased *FGFR1* mRNA levels. This demethylation treatment also significantly activated NF-κB cascades. Methylation analysis showed hypomethylation of the *FGFR1* DNA promoter in asthma samples compared to healthy controls. This study highlights the importance of investigating extracellular RNA and DNA, particularly in understanding the molecular mechanisms underlying asthma. The hypomethylation observed in the extracellular DNA of *FGFR1* suggests that DNA methylation may serve as a crucial regulatory mechanism, influencing gene expression and the progression of inflammatory processes in asthma.

A recent bioinformatics analysis identified *FGFR1* as a potential dysregulated gene in asthma based on consensus gene expression patterns (Loffredo et al., 2017). *FGFR1* plays a central role in modulating epithelial-mesenchymal signaling dysregulation, which may be a shared pathophysiological mechanism among individuals with different asthma severities (Kitai et al., 2016). Wang et al.'s study on lung cancer cell lines revealed elevated *FGFR1* expression, which was associated with the upregulation of genes linked to asthma development, such as ETM-related genes and SOX2 (Wang K. et al., 2018). Notably, our comparison of asthma and healthy samples showed a significant increase in *FGFR1* mRNA levels in asthma samples, supporting the notion of *FGFR1*’s involvement in asthma progression.

NF-ĸB signaling is pivotal in driving various biological processes implicated in developing inflammatory and immune disorders, notably asthma. For instance, its activation in BEAS-2B cells is essential for eliciting adaptive inflammatory responses against intracellular pathogens like PM2.5 and PM1 (Dang et al., 2020). In asthma animal models, inhibition of the NF-ĸB signaling pathway has markedly reduced airway inflammation and remodeling (Chauhan et al., 2018). Conversely, prior research has highlighted *FGFR1* as a proinflammatory factor, activating the NF-ĸB signaling cascade (Wang C. et al., 2018). To delve deeper into the contribution of *FGFR1* in asthma progression, we conducted experiments involving the overexpression of *FGFR1* in BEAS-2B cells to investigate its interplay with NF-ĸB. Consistent with findings by Wang et al. (Reese et al., 2019), our results demonstrated that heightened expression of *FGFR1* significantly enhanced cell proliferation and upregulated NF-ĸB transcription, providing novel insights into the association between *FGFR1* expression and asthma (Bae et al., 2020). Furthermore, epigenetic studies in asthma have underscored the significant role of epigenetic regulation as a primary driver of asthma pathogenesis.

Our findings indicate that *FGFR1* hypomethylation amplifies NF-κB signaling, contributing to the inflammatory processes linked to asthma. This observation is consistent with prior research emphasizing the pivotal role of NF-κB in asthma pathogenesis. By establishing a connection between *FGFR1* methylation and NF-κB activity, our study offers fresh insights into the molecular mechanisms of asthma and proposes potential targets for therapeutic strategies. Subsequent investigations should prioritize *in vivo* models to validate and expand upon these findings. It is important to acknowledge certain limitations of our study. The modest sample size, particularly in the methylation analyses, may compromise the strength of our conclusions, necessitating more extensive studies for validation.

Our findings suggest that DNA methylation of FGFR1 may be a key epigenetic mechanism through which environmental factors influence asthma development (Potaczek et al., 2022). Given that epigenetic modifications are responsive to environmental changes, the observed hypomethylation of FGFR1 in asthma patients could reflect an interaction between genetic susceptibility and environmental exposures. This highlights the potential for targeting epigenetic modifications in therapeutic strategies aimed at mitigating the impact of environmental factors on asthma. Our results showed that using 5-aza-CdR, a broad-spectrum demethylation agent, may lead to the demethylation of multiple loci, potentially impacting the expression of genes beyond *FGFR1*. For instance, 5-aza-CdR has been reported to decrease methylation of the ORMDL3 promoter, thereby promoting inflammation in asthma (Schedel et al., 2015; Toncheva et al., 2015) Polymorphisms related to ORMDL3 are associated with asthma susceptibility. Despite these constraints, our study underscores the potential importance of *FGFR1* demethylation in activating NF-ĸB signaling, which could exacerbate the pathological inflammation associated with asthma. Future investigations, incorporating *in vivo* studies and more targeted epigenetic strategies, are imperative to validate and further elucidate these mechanisms.

## 5 Conclusion

Our findings suggest that extracellular RNA and DNA, particularly through the mechanism of DNA methylation, play a significant role in the regulation of *FGFR1* expression and the activation of NF-ĸB signaling in asthma. These results open new avenues for the development of targeted therapies that focus on epigenetic modifications in extracellular DNA as a means to manage asthma more effectively. Therefore, our study not only underscores the importance of FGFR1 methylation in asthma but also emphasizes the broader role of epigenetic mechanisms in mediating gene-environment interactions, offering new insights into potential intervention strategies. However, we acknowledge that our study lacks detailed clinical and laboratory data, such as asthma phenotypes, allergies, accompanying disorders, asthma severity, treatment, and relevant laboratory tests (e.g., IgE levels, specific IgE/skin prick testing, eosinophilia, spirometry). While these data would have provided additional context and depth to our findings, the focus of our research was on exploring the molecular mechanisms underlying FGFR1 methylation in asthma. Future studies with a more comprehensive clinical dataset are needed to validate and extend our findings. Additionally, in addition to a small number of total plasma samples, the statistical power was insufficient because the number of normal plasma samples was small compared to that for lung cancer patients.

## Data Availability

The original contributions presented in the study are publicly available. This data can be found here https://figshare.com/articles/dataset/Data_Set_Article_ID_1433557/26894038?file=48934777.
