# Supplementary figures and images for: DNA methylation-mediated FGFR1 silencing enhances NF-κB signaling: implications for asthma pathogenesis
Source: Front Mol Biosci. 2024 Sep 23;11:1433557. doi: 10.3389/fmolb.2024.1433557 (PMC11456769; doi:10.3389/fmolb.2024.1433557)

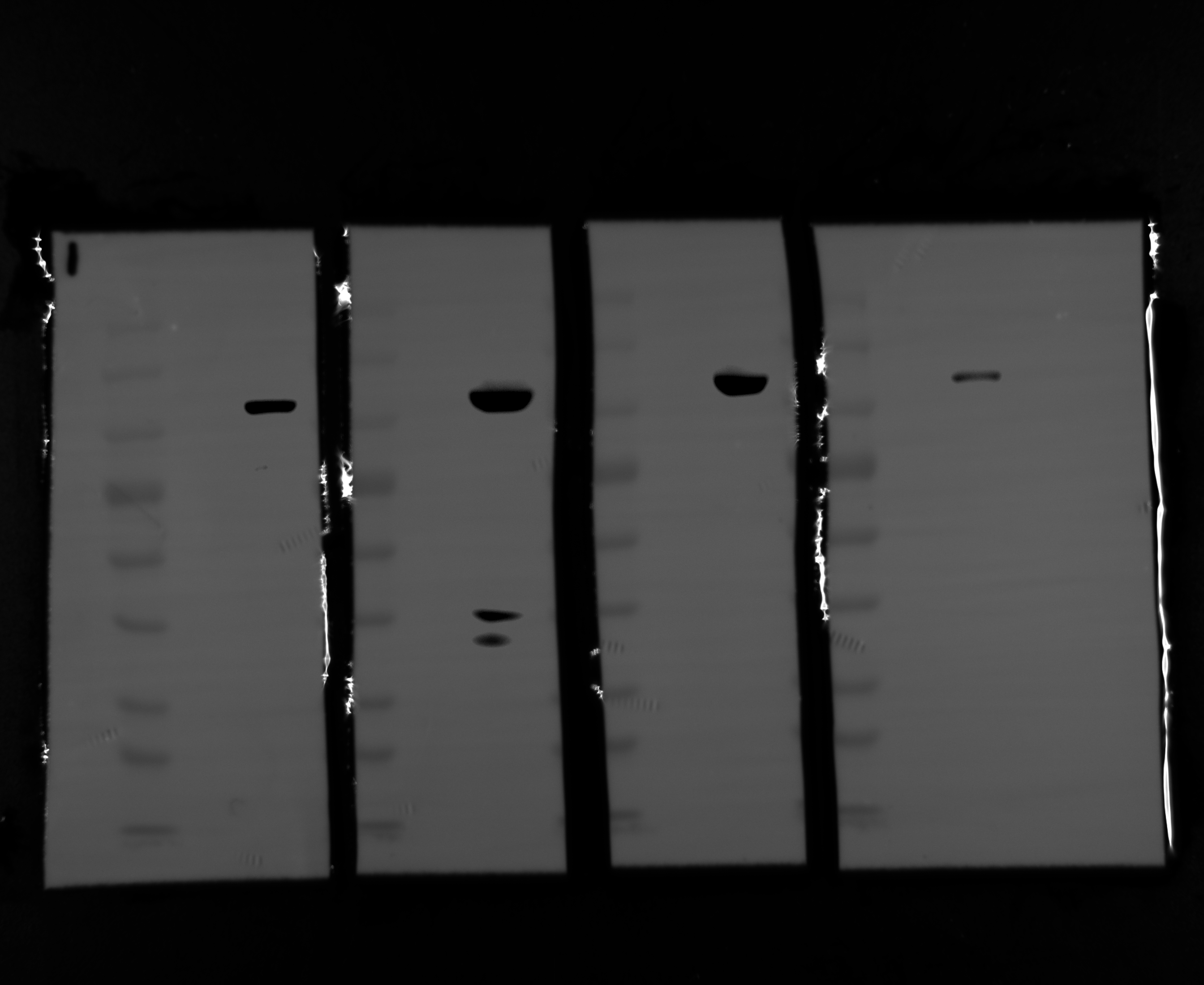

Supplement: Supplementary file 1 [file Image6.TIF]

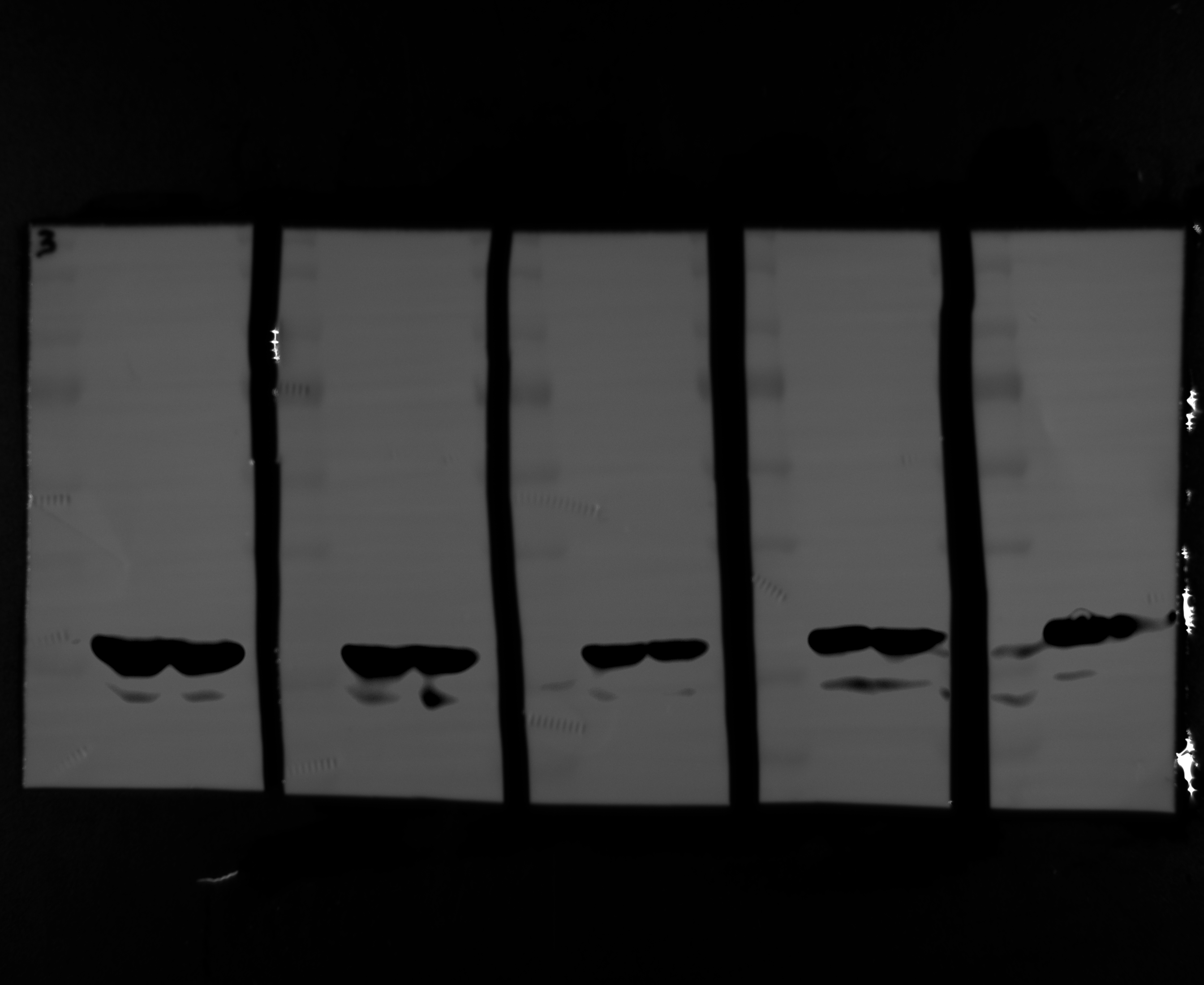

Supplement: Supplementary file 2 [file Image3.TIF]

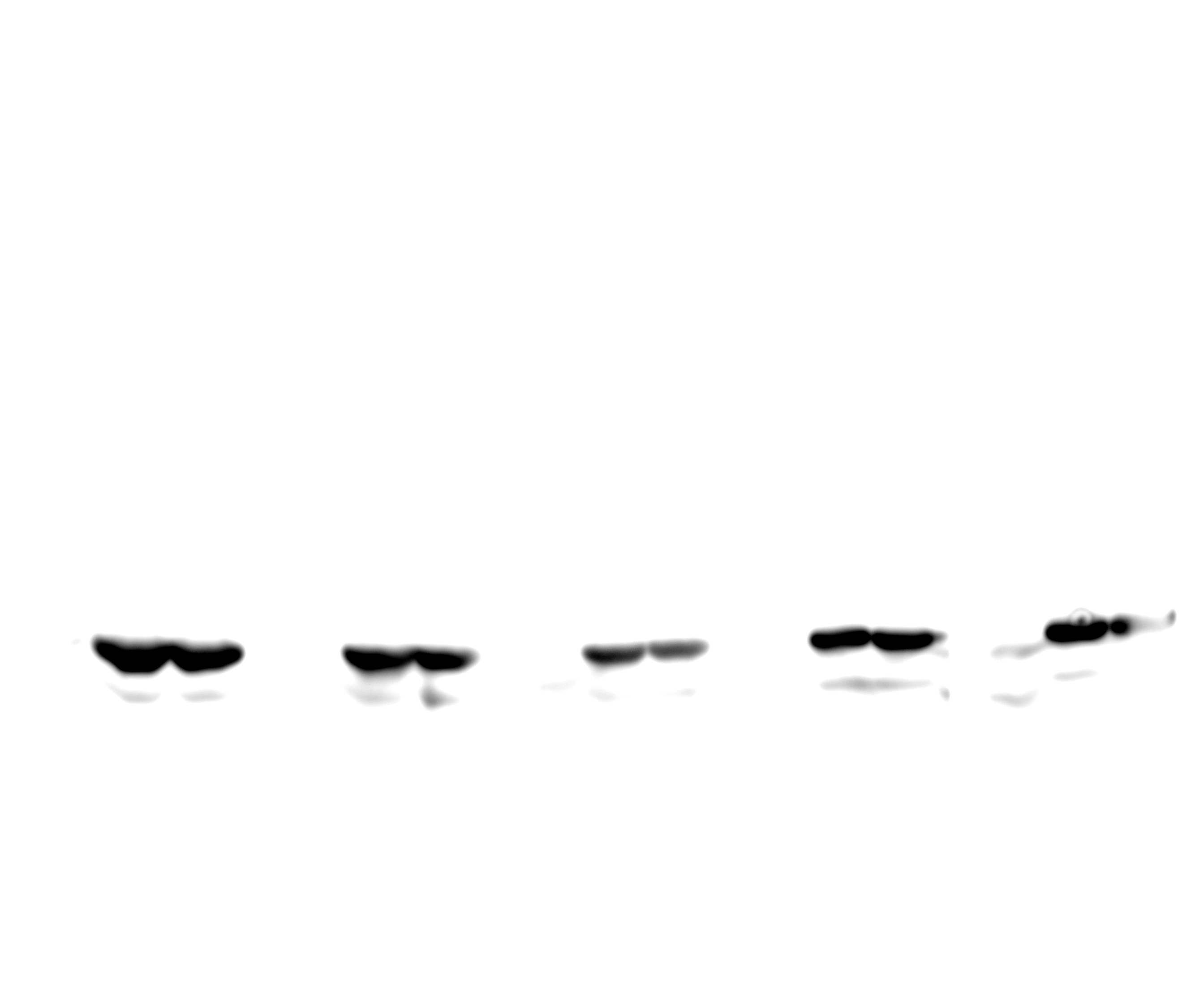

Supplement: Supplementary file 3 [file Image4.TIF]

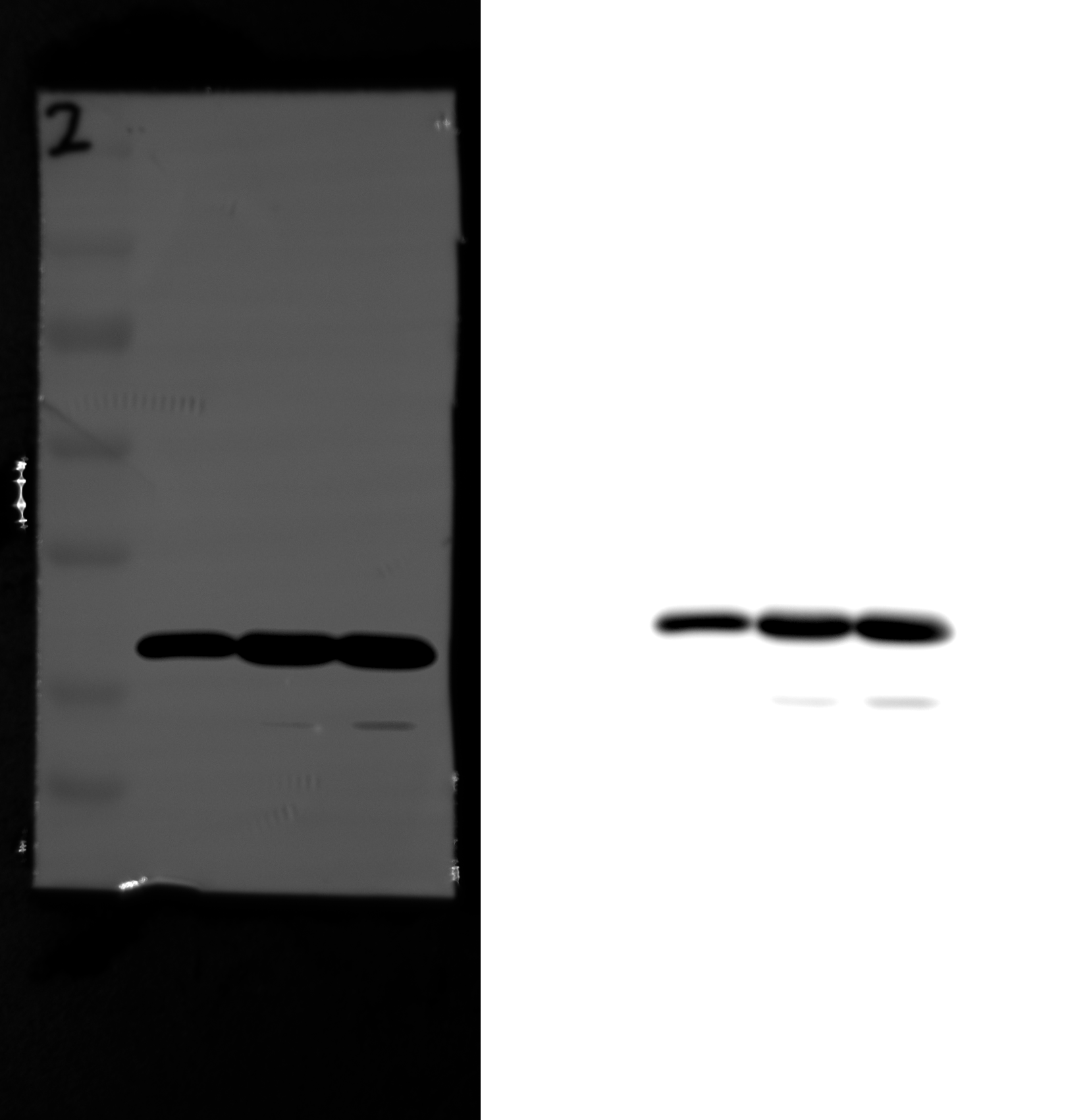

Supplement: Supplementary file 4 [file Image2.TIF]

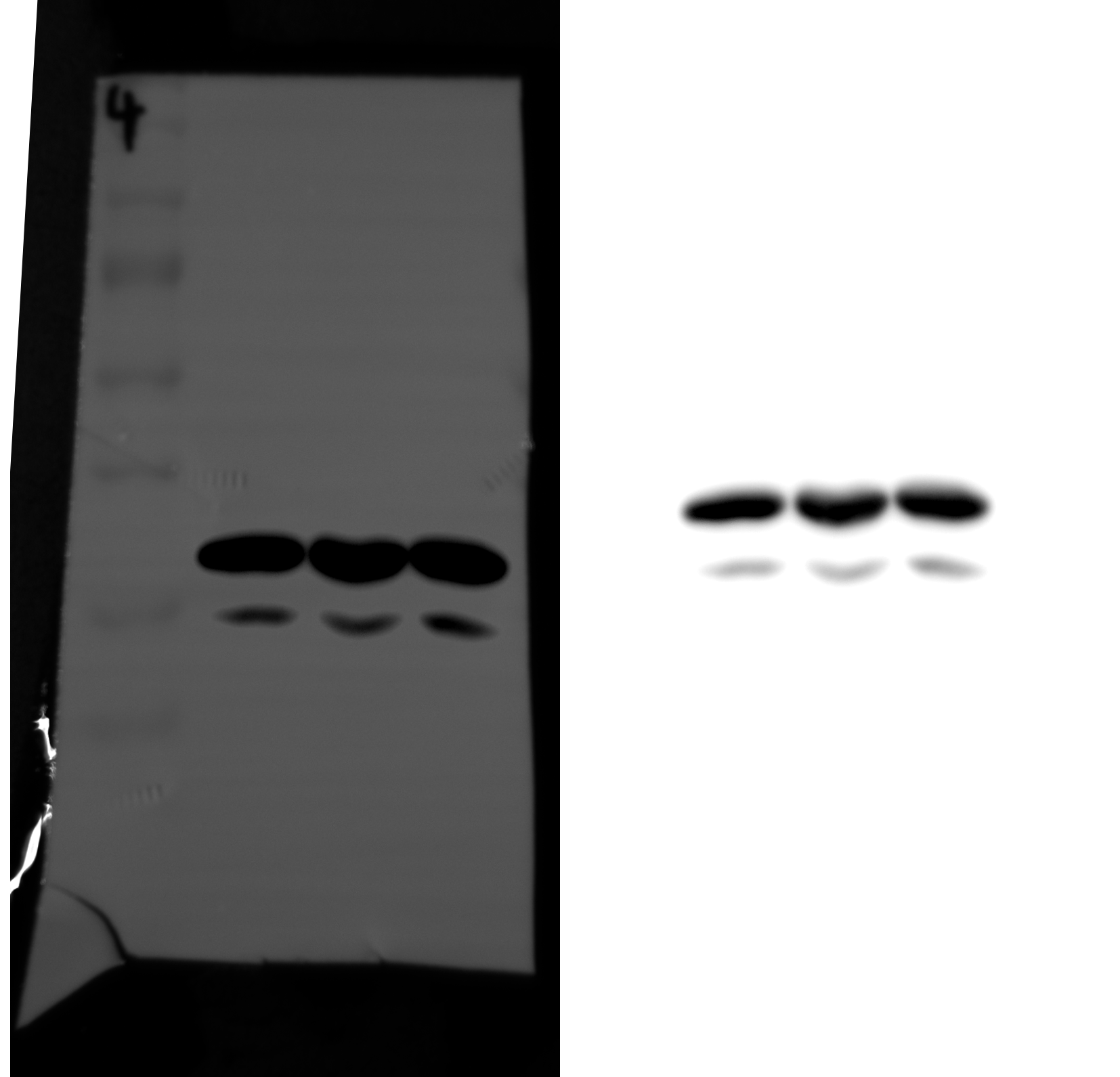

Supplement: Supplementary file 5 [file Image1.TIF]

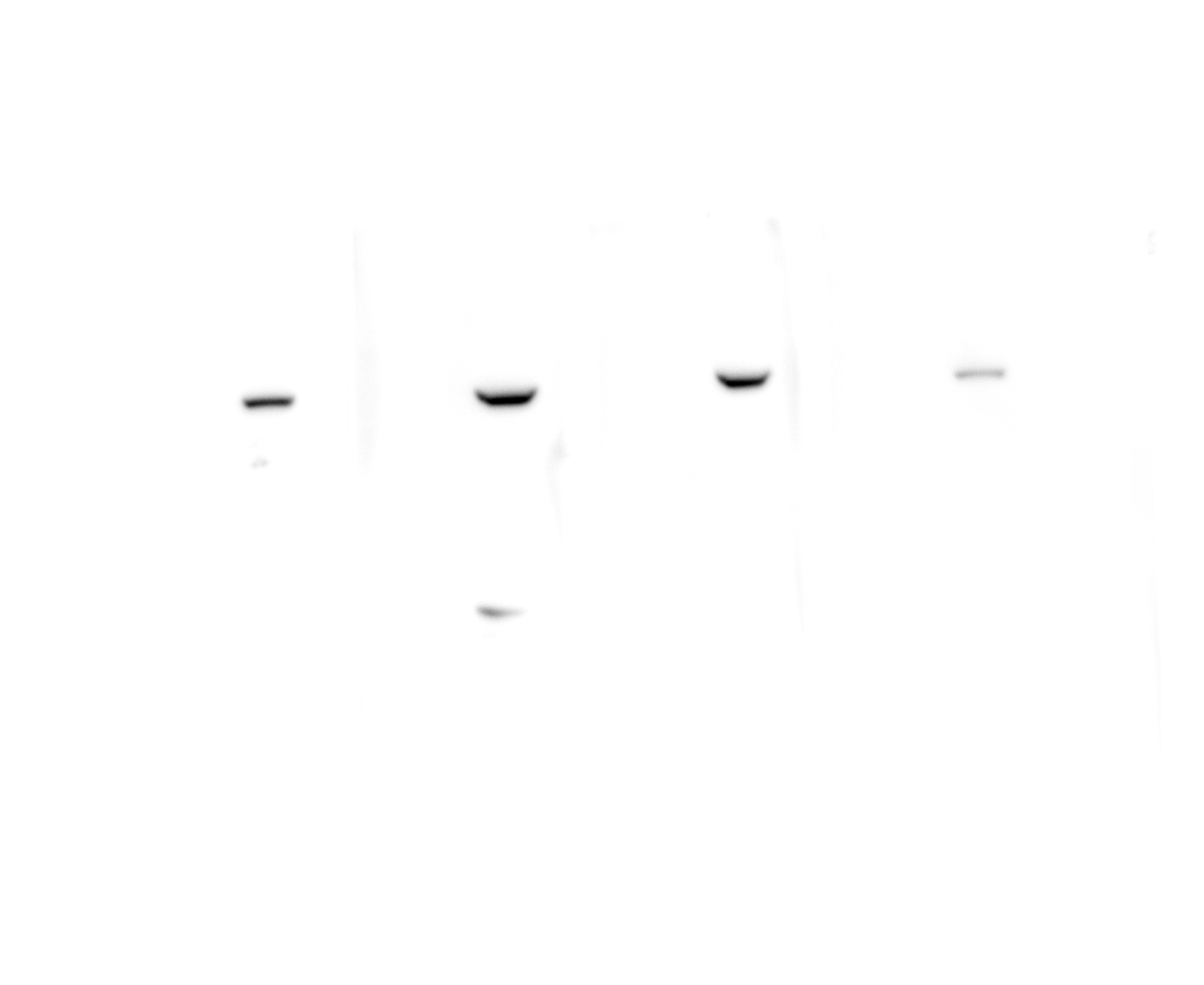

Supplement: Supplementary file 6 [file Image5.TIF]
